# Cesarean delivery rates, costs and readmission of childbirth in the new cooperative medical scheme after implementation of an episode-based bundled payment (EBP) policy

**DOI:** 10.1186/s12889-019-6962-3

**Published:** 2019-05-14

**Authors:** Zhaolin Meng, Kun Zou, Ning Ding, Min Zhu, Yuanyi Cai, Huazhang Wu

**Affiliations:** 1 Department of Health Service Management, School of Humanities and Social Sciences, China Medical University, No.77 Puhe Road, Shenyang, 110122 Liaoning China; 2 Department of Health Policy and Management, West China School of Public Health and Fourth West China Hospital, West China Research Centre for Rural Health Development, Sichuan University, Chengdu, Sichuan China; 3 Institute for International Healthcare Professionals Education and Research, China Medical University, Shenyang, Liaoning China

**Keywords:** Episode-based bundled payment, Cesarean delivery, New cooperative medical scheme, Cost

## Abstract

**Background:**

In the past decade, the rate of cesarean delivery increased dramatically in rural China under the fee-for-service (FFS) system. In September 2011, the New Cooperative Medical Scheme (NCMS) agency in Yong’an county in Fujian province of China adopted a policy of reforming payment for childbirth by transforming the FFS payment into episode-based bundled payment (EBP), which made the cesarean deliveries less profitable. Thus, this study was conducted to determine the effect of EBP policy on reducing cesarean use and controlling delivery costs for rural patients in the NCMS.

**Methods:**

Data from the inpatient information database of the NCMS agency from January 2010 to March 2013 was collected, in which Yong’an county was employed as a reform county and 2 other counties as controls. We investigated the effects of EBP on cesarean delivery rate, costs of childbirth and readmission for rural patients in the NCMS using a natural experiment design and difference in differences (DID) analysis method.

**Results:**

The EBP reform was associated with 33.97% (p<0.01) decrease in the probability of cesarean delivery. The EBP reform, on average, reduced the total spending per admission, government reimbursement expenses per admission, and out-of-pocket (OOP) payments per admission by ¥ 649.61, ¥ 575.01, and ¥ 74.59, respectively. The OOP payments had a net decrease of 14.24% (p<0.01); whereas the OOP payments as a share of total spending had a net increase of 8.72% (p<0.01). There was no evidence of increase in readmission rates.

**Conclusions:**

These results indicate that the EBP policy has achieved at least a short-term success in lowering the increase of cesarean delivery rate and costs of childbirth. Considering both the cesarean rate and the OOP payments as a share of total spending after the reform were still high, China still has a long way to go to achieve the ideal level of cesarean rate and improve the benefits of deliveries for rural population.

**Electronic supplementary material:**

The online version of this article (10.1186/s12889-019-6962-3) contains supplementary material, which is available to authorized users.

## Background

**Fig. 1:**
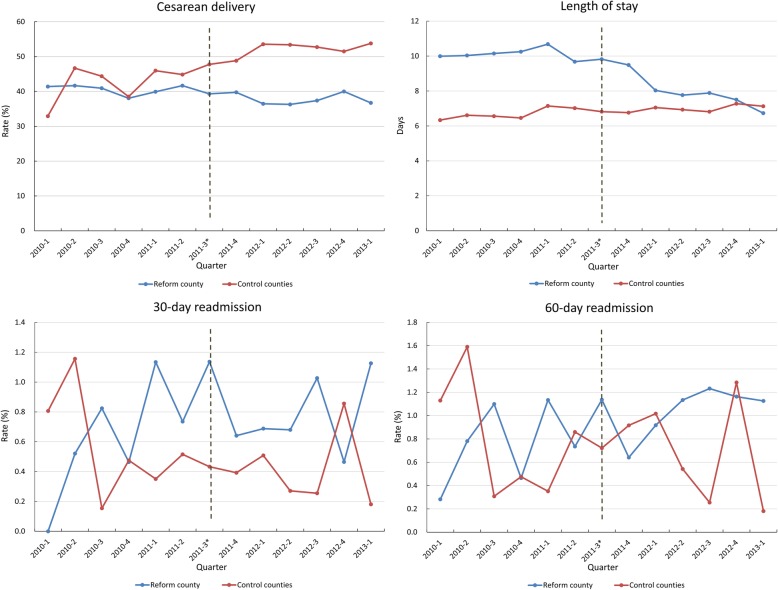
Quarterly time trend of cesarean delivery rate, length of stay and rate of readmission. Note: *The EBP reform was adopted in September 2011

**Fig. 2:**
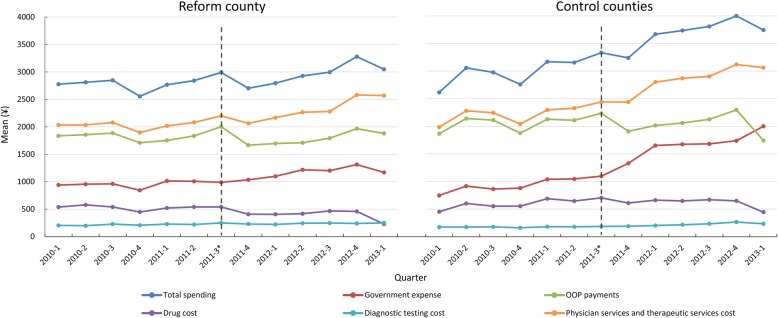
Quarterly time trend of costs of childbirth, by out-of-pocket payments, government expense, type of services. Note: *The EBP reform was adopted in September 2011

The cesarean delivery rate has been a public concern during the past years due to its roaring rising figs. [1, 2]. The cesarean delivery rate in China is significantly higher than that recommended by World Health Organization (WHO) of 15% [3]. Between 1988 and 2008, the cesarean delivery rate increased from 3 to 39%, which initially occurred in urban areas and then in rural areas with the improvement of the health system [4]. A 2008 WHO analysis reported a cesarean rate of 46% in three regions of China [5].

If cesarean deliveries are performed when medically necessary, they are potentially life-saving procedures for women and their babies. However, if women undergo cesarean deliveries without an appropriate medical reason, they are more likely to encounter unnecessary risks, including increased risk of infection, transfusion, injury, anesthetic and surgical complications, and lower likelihood of breast-feeding [6–9]. Unfortunately, in many settings, women are increasingly undergoing cesarean deliveries without a medical indication [10, 11]. A WHO global survey showed that in China, 11.6% of all deliveries were cesareans without a medical indication in 2008 [5]. It is suggested that some factors such as the increased health insurance reimbursement, payment mechanisms and incentives to generate revenues, women’s demands, etc., may have contributed to the high cesarean delivery rate [12–14]. It was found that the increase in cesarean delivery rate in rural China was associated with the reimbursement policy of the New Cooperative Medical Scheme (NCMS) and the revenue-related bonus systems for doctors [12]. Following the demise of the rural Cooperative Medical Scheme (CMS) in the 1980s in the marketization of the rural economy, most rural population were left without health insurance coverage in China. Meanwhile, healthcare facilities relied on user fees to cover their running costs and this resulted in a rapid increase of medical costs. It is claimed that under the fee-for-service (FFS) system of the NCMS, health providers have incentives to perform cesarean deliveries because they were reimbursed with more money, compared with vaginal deliveries [15]. One previous study showed that since the introduction of the NCMS, expenditures on hospital births increased by 152% from 2002 to 2007, with expenses for cesarean delivery accounting for as much as 85% of the total delivery expenses in rural China [15].

Some countries are experimenting with measures to reduce the use of cesareans and control delivery costs [16–18]. According to the Stafford study [16], the measures to decrease cesarean delivery rate were roughly classified into six strategies: (1) education and peer evaluation, (2) external review, (3) medical malpractice reform, (4) changes in physician reimbursement, (5) changes in hospital reimbursement, and (6) public dissemination of cesarean delivery rate. However, evidence on effective ways of achieving this goal among low- and middle- income countries (LMICs), particularly in China, is limited.

In September 2011, the NCMS agency in Yong’an county in Fujian province, southern rural China, adopted a policy reforming payment for childbirth by transforming the FFS payment into episode-based bundled payment (EBP). EBP pays a case rate for an entire episode of care. The EBP, implemented in Yong’an county, is less sophisticated than the diagnosis-related groups (DRGs) based payment system in the United States (US), because there are varied levels of quality in technological capacity and information systems leading to coding difficulties in Chinese hospitals. The DRGs based payment system to classify procedures in the US was thought to be too complicated for routine use in China. Hence a partial implementation of DRGs based payment system utilizing a simpler version was adopted. In Yong’an county, initially cesarean deliveries and vaginal deliveries without complications or complex conditions (amniotic fluid embolization, postpartum hemorrhage, placenta abruption, multiple delivery, etc.) were defined and paid based on the EBP method. The cases with complications or complex conditions were still paid on a FFS basis. Under the EBP system, prices (or tariffs) for uncomplicated cesarean births hospital care were lowered by ¥1752 (from ¥5352 to ¥3600) and the prices for uncomplicated vaginal births were increased by ¥409 (from ¥1591 to ¥2000), which made the cesarean deliveries less profitable. A reliance on reforming payments as a strategy to reduce cesarean delivery assumes that physician decision making is determined, at least in part, by financial incentives. These objectives suggest linkages between the adoption of EBP payment, change in the behavior of decision makers, and subsequent changes in the performance of childbirth care. However, since the EBP policy was introduced in Yong’an, formal evaluation of its impact was rare and of limited utility in guiding decisions for other regions that intend to implement such policy.

This paper presents the findings of the first quantitative empirical analysis of the effects of EBP in Yong’an in its first years of implementation. We investigated the effects of EBP on cesarean delivery rates and costs of childbirth in rural China. It is concerned that fixed price payment systems may compromise quality of care [19, 20]. And, the rate of readmission after discharge is frequently cited as an indicator of quality of care [21]. This study also evaluated whether EBP was associated with the risk of 30-day and 60-day readmission after discharge.

## Methods

### Data and study population

We analyzed data from the NCMS database on all deliveries from January 2010 to March 2013 in three adjacent counties, Yong’an, Sha and Youxi, in the northwest region of Fujian Province. Yong’an county, in which the EBP policy was introduced, was employed as the reform county. Sha county and Youxi county, which had not instituted a payment change for the same period and were similar in their economic development and geographic location, were employed as the control counties. The data from the two counties (Sha and Youxi) were pooled as the control group. As the average level of the two control counties in their offer of obstetric service is comparable to that of the reform county, the cesarean delivery rate within the combined two counties (Sha and Youxi) can be compared with that of the reform county (Yong’an). The characteristics of the reform and control counties are given in Additional file 1.

The study population included 17,322 maternal birth records (identified using the International Statistical Classification of Disease and Related Health Problems, 10th Revision (ICD-10) coding system; O82 and O80 stand for cesarean delivery and vaginal delivery, respectively) that occurred from January 2010 to March 2013 (*N* = 6825 in the reform county, and *N* = 10,497 in the two control counties).

### Study design

We used a natural policy experiment design and a difference in differences (DID) analysis method to estimate average changes in key variables in the reform and control counties before and after the introduction of EBP policy and hence to estimate the average effects of the EBP policy. This DID analysis is a strong quasi-experimental method that has commonly been used to study the effects of policy changes [21]. This statistical method strips out potentially unobserved confounding differences in the reform and control counties that are fixed over time, apart from any that are simultaneous with the implementation of EBP policy. The analysis also controls for differences at baseline. Meanwhile, we performed a sensitivity analysis of DID method with propensity score matching (PSMDID) to examine the robustness of the results.

### Variable measurement

The main dependent variables were the cesarean delivery (Dummy variable = 1 if hospitalized for a cesarean delivery, and 0 otherwise) and the costs of childbirth per admission, including total spending, government reimbursement expenses, OOP payments, OOP payments as a share of total spending and costs of different medical service categories including medications, diagnostic testing, physician services and therapeutic services (e.g., intravenous infusion, acupuncture, and nursing services). All cost variables in the article are converted to 2010 Chinese yuan (¥) using the consumer price index.

Additional dependent variables included length of stay (LOS), 30-day readmission and 60-day readmission. In the NCMS database, each patient has a completely unique health insurance number. These health insurance numbers were used to identify readmission cases by deterministic record linkage within a given period. The validity of each linkage was confirmed by further matching the mother’s date of birth in the 2 records. Thirty-day readmission and 60-day readmission after the initial discharge from the delivery hospitalization were identified, which included all readmissions in any hospital (Dummy variable = 1 if rehospitalized within 30 days or 60 days of discharge, and 0 otherwise).

The three key independent variables included reform indicator (1 for reform county; 0 for control counties), period indicator (1 for the post-policy period from September 2011 to March 2013; 0 for the pre-policy period from January 2010 to August 2011), and their interaction term as the key variable of interest in the DID analysis (Dummy variable = 1 if hospitalized after September 2011 in a reform county; and it equals 0 before September 2011 in a reform county; it equals 0 for all years if hospitalized in the control counties).

The controlled variables included key patient characteristics: age (Age and Age^2^, to account for nonlinear effects), poverty level (Dummy variable = 1 if patient was from a poverty-stricken family, defined as the one with annual income under the poverty line in China and with financial aid from the central government; 0 otherwise), and whether the patient was the head of the household (Dummy variable = 1 if patient was the head of the household, and 0 otherwise). Head of household is defined as the head of the householders in the household registration system in China. Head of household is a measure of a family member’s status, and it often acts as a key decision maker in healthcare seeking [22]. Given the existing differences in charging standard and reimbursement ratio between tertiary hospitals and non-tertiary hospitals, the hospital level was also brought into controlled variables (1 = hospitalized in a tertiary hospital; 0 = hospitalized in a non-tertiary hospital). In Chinese hospitals’ classification criterion, tertiary hospitals have more than 500 beds [23].

### Statistical analyses

DID method was used to compare changes of key indicators before-after the payment reform between reform county and control counties, controlling for other relevant factors [21]. Specifically, we use the linear regression fitted by the least square approach with the following empirical specification (1):

1 $$ {\displaystyle \begin{array}{l}\mathrm{Y}i=\beta 0+\beta 1\operatorname{Re}\mathrm{form}i+\beta 2\mathrm{Period}i+\beta 3\operatorname{Re}\mathrm{form}i\ast \mathrm{Period}i+\beta 4\mathrm{Age}i+\beta 5{\left(\mathrm{Age}i\right)}^2\\ {}+\beta 6\mathrm{Household}i+\beta 7\mathrm{Poverty}i+\beta 8\mathrm{Tertiary}i+\varepsilon \end{array}} $$

Y_*i*_ represents the dependent variables: cesarean delivery, costs indicators, as well as LOS and readmission. The coefficient *β*1 captures time-independent difference between reform county and control counties, *β*2 captures the difference in ‘before’ and ‘after’ the policy was implemented and *β*3 estimates the difference in differences between reform county and control counties in ‘before’ and ‘after’ the policy was implemented. The coefficients from *β*4 to *β*8 represent a series of covariates, including age, age^2^, whether the patient was the head of the household, poverty level and whether the patient was hospitalized in a tertiary hospital. Age was centered (=calendar age − mean age in the sample) before computing the quadratic age term to reduce issues of multicollinearity [24]. ε refers to the error term. The variance inflation factor for all variables used in the models was found to be < 4.0, indicating no multicollinearity problems.

Analyses were conducted using SPSS (version 25.0) and SAS (version 9.3). SPSS was used for propensity score matching, while SAS was used for all other statistical analyses. Statistical significance was based on 2-sided tests and accepted at the *p* ≤ 0.05 level of significance.

## Results

### Study sample characteristics

As expected with a large sample size, nearly all covariates showed significant differences between the reform county and control counties (Table 1). Substantively meaningful differences were more limited, but reflected differences in characteristics of the deliveries in the reform county compared with control counties. For example, the reform county had higher percentage of patients who were the head of the household and higher percentage of patients hospitalized in tertiary hospitals, compared to the control counties. The patients in the reform county (26.50 years) were slightly older than that in the control counties (26.05 years).

**Table 1:** Summary characteristics of patients in 2010 before the payment reform implementation

	Reform county (*n* = 3269)	Control counties (*n* = 5108)	X^2^/t	p
Age, years	26.50 [4.36]	26.05 [4.32]	4.664	0.000^**^
Head of household^a^, %	2.4	1.2	20.397	0.000^**^
From the poverty-stricken family^b^, %	1.8	1.9	0.227	0.634
Hospitalized in a tertiary hospital,%	24.4	18.5	42.088	0.000^**^

^**^p<0.01. S.D. are in square brackets

^a^Head of household: defined as the head of the householders in the household registration system in China

^b^Poverty-stricken family: defined as the one with annual income under the poverty line in China and with financial aid from the central government

### Quarterly time trend of cesarean delivery rates, costs, LOS and readmission rates

Figure 1 shows quarterly time trend of unadjusted cesarean delivery rates, LOS and rates of readmission. After EBP reform, the cesarean delivery rates and LOS decreased considerably in the reform county, but they increased or remained similar in the control counties. There was no considerable change of readmission rate in the two groups after the reform.

Figure 2 presents quarterly unadjusted costs of childbirth per admission. Overall, total spending, the government reimbursement amount and costs of different medical service categories in the reform county presented a small decline or increased gradually after EBP reform compared with the sharp rise in the control counties.

### Policy effects on cesarean deliveries, costs, LOS and readmissions

As shown in Table 2, cesarean delivery rates in the reform county increased by 2.4% (from 35.5 to 37.9%) after the reform, while that of the control counties grew by 12.5% (from 39.0 to 51.5%), leading to a DID estimate of the impact of EBP reform of − 10.1%. Total spending, government reimbursement expenses and costs of different medical service categories showed sizable DID cost reductions. The EBP reform, on average, reduced the total spending per admission, government reimbursement expenses per admission, and OOP payments per admission by ¥ 649.61, ¥ 575.01, and ¥ 74.59, respectively. OOP payments as a share of total spending in the reform county decreased by 3.2% (from 62.82 to 59.62%) after the reform, while that of the control counties decreased by 11.62% (from 68.90 to 57.28%), leading to a DID estimate of the impact of EBP reform of 8.41%. LOS decreased by 1.74 days associated with the EBP reform. There was no significant difference of change of 30-day and 60-day readmissions (*p* > 0.05).

**Table 2:** Impact of payment reform on cesarean delivery rate, costs of childbirth per admission, length of stay and rate of readmission: average estimates

	Reform county			Control counties			DID
	Pre	Post	Diff	Pre	Post	Diff	
Cesarean delivery rate, %	35.50	37.90	2.40^*^	39.00	51.50	12.50^**^	−10.10
Total spending (¥)	2847.42 [2295.65]	3023.49 [2606.99]	176.07^**^	2886.27 [1894.56]	3711.95 [2040.98]	825.68^**^	−649.61
Government expenses (¥)	955.24 [709.99]	1181.57 [1139.67]	226.33^**^	889.59 [709.25]	1690.93 [1201.82]	801.34^**^	−575.01
OOP^a^ payments (¥)	1892.18 [1654.14]	1841.92 [1643.38]	−50.25	1996.68 [1372.69]	2021.02 [1242.78]	24.34	−74.59
OOP^a^ payments as a share of total spending,%	62.82 [9.64]	59.62 [10.03]	−3.20^**^	68.90 [14.10]	57.28 [18.17]	−11.62^**^	8.41
Drug cost (¥)	536.26 [684.09]	377.12 [616.97]	−159.14^**^	559.02 [667.99]	594.07 [662.95]	35.05^**^	− 194.19
Diagnostic testing cost (¥)	225.41 [226.74]	246.13 [275.13]	20.72^**^	176.95 [171.13]	225.24 [214.23]	48.29^**^	−27.57
Physician services and therapeutic services cost (¥)	2085.75 [1482.30]	2400.24 [1839.96]	314.50^**^	2150.31 [1243.58]	2892.64 [1449.86]	742.34^**^	− 427.84
LOS^b^, days	9.57 [5.92]	7.85 [4.63]	−1.72^**^	6.58 [2.82]	7.00 [3.01]	0.02^**^	−1.74
30 day readmission rate, %	0.60	1.00	0.40	0.50	0.50	0.00	0.40
60 day readmission rate, %	0.80	1.30	0.50	0.70	0.90	0.20	0.30

‘Pre’ is January 2010–August 2011; ‘Post’ is September 2011–March 2013; Diff is after minus before; DID is diff for reform county minus diff for control counties. The number of observations is 3269 in January 2010–August 2011 and 3556 in September 2011–March 2013 for reform county, and 5108 in January 2010–August 2011 and 5389 in September 2011–March 2013 for control counties. S.D. are in square brackets. ^a^OOP: Out-of-pocket; ^b^LOS: Length of stay. ^*^p<0.05; ^**^p<0.01

According to the results of multivariate regressions in Table 3, the EBP reform was associated with a 33.97% (=1-EXP^-0.4150^, p<0.01) decrease in the probability of cesarean delivery. Meanwhile, the reform reduced total spending (31.46%, p<0.01), government expenses (60.3%, p<0.01), and costs of medications (86.43%, p<0.01), diagnostic testing (20.7%, p<0.01), physician services and therapeutic service (25.82%, p<0.01). The OOP payments had a net decrease of 14.24% (p<0.01); but the OOP payments as a share of total spending had a net increase of 8.72% (p<0.01). The reform decreased the LOS by 17.29% (p<0.01). The results of 30-day and 60-day readmissions were not significant (p > 0.05).

**Table 3:** Multivariate regression DID estimates of the impact of payment reform on cesarean delivery, costs of childbirth per admission, length of stay and readmission

Variable	Cesarean delivery	Ln (Total spending)	Ln (Government expenses)	Ln (OOP^a^ payments)	OOP^a^ % of total spending	Ln (Drug cost)	Ln (Diagnostic testing cost)	Ln (Other cost^b^)	Ln (LOS^c^)	30-day readmission	60-day readmission
Reform*Period	−0.4150^**^ (0.0645)	− 0.3146^**^ (0.0210)	− 0.6030^**^ (0.0264)	− 0.1424^**^ (0.0217)	8.7236^**^ (0.4038)	− 0.8643^**^ (0.0646)	− 0.2070^**^ (0.0268)	− 0.2582^**^ (0.0187)	− 0.1729^**^ (0.0150)	0.400 (0.3921)	0.2772 (0.3312)
Reform	− 0.1576^*^ (0.0468)	− 0.1270^**^ (0.0152)	0.1737^**^ (0.0191)	0.2216^**^ (0.0156)	−6.8458^**^ (0.2909)	− 0.5569^**^ (0.0459)	0.1526^**^ (0.0189)	− 0.1118^**^ (0.0135)	0.2252^**^ (0.0109)	0.1617 (0.2990)	0.0279 (0.2605)
Period	0.5207^**^ (0.0399)	0.3036^**^(0.0132)	0.6941^**^ (0.0166)	0.0651^**^ (0.0137)	−12.5595^**^ (0.2539)	0.4665^**^ (0.0393)	0.2108^**^ (0.0164)	0.3257^**^ (0.0118)	0.0554^**^ (0.0093)	−0.0081 (0.2715)	0.2071 (0.2176)
Age	0.0415^**^ (0.0046)	0.0145^**^(0.0015)	0.0145^**^ (0.0019)	0.0148^**^ (0.0015)	0.0135 (0.0286)	0.0281^**^ (0.0045)	0.0008^**^ (0.0019)	0.0128^**^ (0.0013)	0.0047^**^ (0.0011)	0.0250 (0.0323)	0.0083 (0.0256)
Age^2^	0.0002 (0.0006)	−0.0001 (0.0002)	0.0003 (0.0003)	−0.0003 (0.0002)	− 0.0105^**^ (0.0041)	−0.0001 (0.0006)	0.0002 (0.0003)	−0.0001 (0.0002)	− 0.000006 (0.0002)	−0.0232^**^ (0.0075)	− 0.0151^**^ (0.0054)
Household	0.1181 (0.1107)	0.0050 (0.0365)	0.0540 (0.0458)	−0.0173 (0.0376)	−1.3474 (0.6998)	−0.0453 (0.1105)	0.0319 (0.0463)	−0.0010 (0.0324)	0.0131 (0.0263)	−0.2862 (0.7209)	− 0.1916 (0.5903)
Poverty	0.4256^*^ (0.1117)	0.1303^**^ (0.0372)	0.1409^**^ (0.0468)	0.1305^**^ (0.0383)	0.0298 (0.7132)	0.3559^**^ (0.1116)	0.1603^**^ (0.0468)	0.1010^**^ (0.0330)	0.0734^**^ (0.0262)	−0.0418 (0.7166)	0.5051 (0.4587)
Tertiary	−0.2507^**^ (0.0372)	0.3339^**^ (0.0121)	−0.0781^**^ (0.0152)	0.5562^**^ (0.0125)	13.1980^**^ (0.2319)	0.5286^**^ (0.0364)	0.7678^**^ (0.0151)	0.2575^**^ (0.0107)	0.0797^**^ (0.0086)	0.363 (0.2073)	0.4586^**^ (0.1696)
N	17,311	17,310	17,297	17,310	17,310	16,578	16,598	17,310	16,894	17,311	17,311

Standard errors in parentheses. ^*^p<0.05; ^**^p<0.01. ^a^*OOP* Out-of-pocket; ^b^Other cost: Physician services and therapeutic services cost; ^c^*LOS* Length of stay, *DID* Difference in differences

### Robustness

We conducted the PSMDID to eliminate the observable differences between the reform county and the control counties. The estimated results of PSMDID are shown in Additional file 2. We observed that the estimated coefficients of PSMDID were quite similar to the results of our main DID analyses. These results implied that the results of DID in our study were robust.

## Discussion

Our study showed that cesarean delivery rate and costs of childbirth had sizable DID reductions associated with the EBP policy, while the OOP payments as a share of total spending had a significant net increase. The reform led to a reduction of LOS, while there was no evidence of increase in readmission rates. These results suggested the EBP reform having the potential for reducing cesarean use and controlling delivery costs for the rural population.

Consistent with theoretical predictions, the combined policies of making cesarean delivery less profitable and making the uncomplicated vaginal births more profitable created an incentive for vaginal delivery, which led to a significant net decrease of the cesarean delivery rates. However, the cesarean delivery rate after the reform in the reform county was still more than 30%, far higher than the WHO recommendation of 15% [3]. Previous studies showed that high cesarean delivery rate could be ascribed to a range of interrelated provider, patient, and health system factors [25]. Besides the effect of payment reform on cesarean delivery rate, there are still other related factors. An important patient-related factor: the rising prevalence of overweight and obesity, exists in rural China, which may link to the high cesarean delivery rate. Unfortunately, the data about Body Mass Index (BMI) is not available in our study, but previous studies have shown that the prevalence of obesity has increased dramatically in rural China over the last decades [26, 27]. In 2016, a study specifically investigated the distribution of BMI among women of reproductive age before pregnancy in rural China, showed that the prevalence of overweight and obesity was 24.8% for adult women and 17.2% for adolescent girls [26]. The epidemic of excess weight in rural women and adolescent girls is associated with remarkable changes in living environments, nutrition transition, and sedentary lifestyles owing to rapid economic growth and urbanization in China [27, 28]. An increased risk of cesarean delivery in obese patients has been repeatedly demonstrated [29–31]. Future studies should focus on the prevalence of obesity in their samples and investigate if emphasis on weight control for women of reproductive age before pregnancy could be a useful tool to reduce the unnecessary cesarean deliveries.

The reform reduced total spending, medications, diagnostic testing, physician services and therapeutic services of childbirth by 31.46%(p<0.01), 86.43%(p<0.01), 20.7%(p<0.01) and 25.82%(p<0.01), respectively. It has been claimed that because of distorted provider incentives in the FFS system, irrational use of medicines and tests is a serious problem in China [32]. Under the EBP system in Yong’an, hospitals can keep any savings but also take financial risk of any cost overrun for hospital patients. This risk sharing is an incentive for hospitals to reduce the unnecessary services, as a strategy for containing costs. By reducing the costs of different medical service categories, total spending was reduced.

It is noteworthy that although both the government expenses and OOP payments had a net decrease, the government expenses were lowered in a much bigger magnitude, compared with OOP payments (¥ 575.01 vs. ¥ 74.59); which revealed that the savings of government expenses mainly contributed to the costs slowdown. And, as seen in this study, OOP payments as a share of total spending had a significant net increase, which implied cost shifting to patients may exist. Under the EBP system in Yong’an, some services and medical consumables for deliveries, such as newborn screening items, absorbable suture and anesthesia analgesic pump were not covered by the NCMS, and therefore, patients had to pay 100%. Previous studies showed that the suppliers may shift their focus on OOP payments which is out of the jurisdiction of the prepaid system to make up for the loss caused by control of the insurance fund, thus they may select items outside of the reimbursement list, leading to the increase of the OOP payments [33, 34]. This may explain why the OOP payments as a share of total spending had a net increase. This result suggests that policymakers should be alert to take effective measures to prevent cost shifting to patients. It is also important to note that OOP payments as a share of total spending after the reform was still more than 50%, higher than that in high-income countries which is generally below 30% [35]; China still has a long way to go to improve the delivery benefits rural people have enjoyed from the NCMS.

All payment methods generate a mix of incentives, requiring mitigation of adverse effects. In the EBP system, a prospective fixed payment rate may make physicians circumvent payment control and maximize profit, such as increasing the number of readmissions through reducing the LOS and increasing premature discharges and shifting care to outpatient departments [36]. The reform led to a reduction of LOS in this study, while we did not find evidence of significant increase in hospital readmissions by the DID results. Meanwhile, deliveries remained on an inpatient basis, and thus, there are no concerns regarding patient selection bias among patients treated inside or outside the hospitals.

### Policy implications

The unhealthy rise in global cesarean delivery rates creates a need to decrease the unnecessary cesarean deliveries and control costs to reduce the financial burden on a delivery, especially for vulnerable populations, such as rural populations [2, 5]. These strategies of EBP reform in China may be of particular interest to other LMICs with the similar issue. Many LMICs are also experiencing remarkable increases of cesarean delivery procedures [5, 11], and these countries also have varied levels of quality in technological capacity that are important for the smooth implementation of DRGs based payment [36]. These countries have the potential to use EBP for uncomplicated delivery procedures. Although a simplified DRGs based payment method that allows some cases with severe complications still to be paid on a FFS basis in the EBP system can hinder the full realization of cost savings and other purposes of policy, this method may be an effective and feasible practice, given the lack of information and technology needed in the DRGs classification system in these countries, especially in a transition period.

### Limitations

There are limitations to this study. Firstly, we only used 1.5 years of data after the EBP reform for our analysis, these findings cannot be interpreted as long-term effects of the reform. Secondly, since we couldn’t obtain data about the quality of care such as in-hospital mortality, although there was no evidence of increase in readmission rates, it is not for certain whether the reform had reduced the quality of care in the long-term. Quality of care should be closely monitored in the future. Thirdly, policies may have differential effects among subgroups. Changes in the population with NCMS-funded births have the potential to introduce unmeasured confounding if the changes occurred at the time of implementation of the payment policy. Although we controlled for some characteristics where available, the nature of the data limits the inclusion of all possible factors. Variables such as education level and BMI were some of the plausible factors that were not included in the NCMS database. However, according to the nine-year compulsory education policy of China’s Ministry of Education and the reality that the majority of rural residents do not have a higher education in China, it would be reasonable to assume that most people in the three counties had a similar secondary education level. Moreover, there was no significant policy change in education in the three counties during the study periods. Therefore, we assume that the education level in this sample group may have minor effects for our analysis. In addition, the data about BMI of this sample is also not available in our study, and thus, this facet would need further study. Considering there are some limitations, these findings, therefore, need to be interpreted cautiously.

## Conclusions

Despite these limitations, our study provides evidence that the EBP policy implications helped to lower the cesarean delivery rates and the costs of childbirth, at least in its first years of implementation. Considering both the cesarean rates and the OOP payments as a share of total spending after the reform were still high, China has a long way to go to achieve the ideal level of cesarean delivery rates and improve the benefits of those deliveries for the rural population.

## Additional files

Additional file 1: Basic information of reform county (Yong’an) and control counties (Sha and Youxi) (2013). (DOC 44 kb)

Additional file 2: The estimated results of difference in differences with propensity score matching. (DOC 47 kb)

## Abbreviations

BMI: Body Mass Index
CMS: Cooperative Medical Scheme
DID: Difference in differences
DRGs: Diagnosis-related groups
EBP: Episode-based bundled payment
FFS: Fee-for-service
ICD-10: International Statistical Classification of Disease and Related Health Problems, 10th Revision
LMICs: Low- and middle-income countries
LOS: Length of stay
NCMS: New Cooperative Medical Scheme
OOP: Out-of-pocket
PSMDID: Difference in differences with propensity score matching
US: United States
WHO: World Health Organization

## References

[1] Hopkins K (2000). Are Brazilian women really choosing to delivery by cesarean?. Soc Sci Med.

[2] Robson SJ, De Costa CM (2017). Thirty years of the World Health Organization's target cesarean section rate: time to move on. Med J Aust.

[3] World Health Organization (2015). WHO statement on cesarean section rates. Department of Reproductive Health and Research.

[4] Hellerstein S, Feldman S, Duan T (2015). China’s 50% cesarean delivery rate: is it too high?. BJOG Int J Obstet Gynaecol.

[5] Lumbiganon P, Laopaiboon M, G€ulmezoglu AM, Souza JP, Taneepanichskul S, Ruyan P (2010). Method of delivery and pregnancy outcomes in Asia: the WHO global survey on maternal and perinatal health 2007–08. Lancet..

[6] Bodner K, Wierrani F, Grünberger W, Bodner-Adler B (2010). Influence of the mode of delivery on maternal and neonatal outcomes: a comparison between elective cesarean section and planned vaginal delivery in a low-risk obstetric population. Arch Gynecol Obstet.

[7] Gregory K, Jackson S, Korst L, Fridman M (2012). Cesarean versus vaginal delivery: whose risks? Whose benefits?. Am J Perinatol.

[8] Pallasmaa N, Ekblad U, Aitokallio-Tallberg A, Uotila J, Raudaskoski T, Ulander VM (2010). Cesarean delivery in Finland: maternal complications and obstetric risk factors. Acta Obstet Gynecol Scand.

[9] Quiroz LH, Chang H, Blomquist JL, Okoh YK, Handa VL (2009). Scheduled cesarean delivery: maternal and neonatal risks in primiparous women in a community hospital setting. Am J Perinatol.

[10] Villar J, Valladares E, Wojdyla D, Zavaleta N, Carroli G, Velazco A (2006). Cesarean delivery rates, pregnancy outcomes: the 2005 WHO global survey on maternal and perinatal health in Latin America. Lancet..

[11] Betrán AP, Merialdi M, Lauer JA, Bing-Shun W, Thomas J, Van Look P (2007). Rates of cesarean section: analysis of global, regional and national estimates. Paediatric and perinatal epidemiology.

[12] Bogg L, Huang K, Long Q, Shen Y, Hemminki E (2010). Dramatic increase of cesarean deliveries in the midst of health reforms in rural China. Soc Sci Med.

[13] Tang S, Li X, Wu Z (2006). Rising cesarean delivery rate in primiparous women in urban China: evidence from three nationwide household health surveys. Am J Obstet Gynecol.

[14] Gleicher N (1984). Cesarean section rates in the United States: the short-term failure of the National Consensus Development Conference in 1980. JAMA..

[15] Long Q, Zhang Y, Raven J, Wu Z, Bogg L, Tang S (2011). Giving birth at a health care facility in rural China: is it affordable?. Bull World Health Organ.

[16] Stafford RS (1990). Alternative strategies for controlling rising cesarean section rates. JAMA..

[17] Liu S, Wang J, Zhang L, Zhang X (2018). Cesarean section rate and cost control effectiveness of case payment reform in the new cooperative medical scheme for delivery: evidence from Xi County, China. BMC Pregnancy Childbirth.

[18] Kozhimannil KB, Graves AJ, Ecklund AM, Shah N, Aggarwal M, Snowden JM (2018). Cesarean delivery rates and costs of childbirth in a state Medicaid program after implementation of a blended payment policy. Med Care.

[19] Chen LM, Ryan AM, Shih T, Thumma JR, Dimick JB (2018). Medicare's acute care episode demonstration: effects of bundled payments on costs and quality of surgical care. Health Serv Res.

[20] Wilner S, Schoenbaum SC, Monson RR, Winickoff RN (1981). A comparison of the quality of maternity care between a health-maintenance organization and fee-for-service practices. N Engl J Med.

[21] Yip W, Eggleston K (2001). Provider payment reform in China: the case of hospital reimbursement in Hainan province. Health Econ.

[22] Vallieres F, Hansen A, McAuliffe E, Cassidy EL, Owora P, Kappler S (2013). Head of household education level as a factor influencing whether delivery takes place in the presence of a skilled birth attendant in Busia, Uganda: a cross-sectional household study. BMC Pregnancy and Childbirth.

[23] Yip WCM, Hsiao W, Meng QY, Chen W, Sun XM (2010). Realignment of incentives for health-care providers in China. Lancet..

[24] Van der Elst W, Hurks P, Wassenberg R, Meijs C, Jolles J (2011). Animal verbal fluency and design fluency in school-aged children: effects of age, sex, and mean level of parental education, and regression-based normative data. J Clin Exp Neuropsychol.

[25] Colomar M, Cafferata ML, Aleman A, Castellano G, Elorrio EG, Althabe F (2014). Mode of childbirth in low-risk pregnancies: Nicaraguan physicians' viewpoints. Matern Child Health J.

[26] He Y, Pan A, Yang Y, Wang Y, Xu J, Zhang Y (2016). Prevalence of underweight, overweight, and obesity among reproductive-age women and adolescent girls in rural China. Am J Public Health.

[27] Huang C, Yu H, Koplan JP (2014). Can China diminish its burden of non-communicable diseases and injuries by promoting health in its policies, practices, and incentives?. Lancet..

[28] Yang G, Wang Y, Zeng Y, Gao GF, Liang XF, Zhou MG (2013). Rapid health transition in China, 1990–2010: findings from the Global Burden of Disease Study 2010. Lancet.

[29] Weiss JL, Malone FD, Emig D, Ball RH, Nyberg DA, Comstock CH (2004). Obesity, obstetric complications and cesarean delivery rate-a population based screening study. Am J Obstet Gynecol.

[30] Beyer DA, Amari F, Lüdders DW, Diedrich K, Weichert J (2011). Obesity decreases the chance to deliver spontaneously. Arch Gynecol Obstet.

[31] Mission JF, Marshall NE, Caughey AB (2013). Obesity in pregnancy: a big problem and getting bigger. Obstetrical & Gynecological Survey.

[32] Hu S, Tang S, Liu Y, Zhao Y, Escobar ML, De Ferranti D (2008). Reform of how health care is paid for in China: challenges and opportunities. Lancet..

[33] Park JD, Kim E, Werner RM (2015). Inpatient hospital charge variability of US hospitals. J Gen Intern Med.

[34] He Ruibo, Miao Yudong, Ye Ting, Zhang Yan, Tang Wenxi, Li Zhong, Zhang Liang (2017). The effects of global budget on cost control and readmission in rural China: a difference-in-difference analysis. Journal of Medical Economics.

[35] World health organization: new perspectives on global health spending for universal health coverage. http://www.who.int/health_financing/topics/resource-tracking/new-perspectives/en/. Accessed 16 Jan 2018.

[36] Quinn K (2014). After the revolution: DRGs at age 30. Ann Intern Med.

