# Supplementary material for: Cesarean delivery rates, costs and readmission of childbirth in the new cooperative medical scheme after implementation of an episode-based bundled payment (EBP) policy
Source: BMC Public Health. 2019 May 14;19:557. doi: 10.1186/s12889-019-6962-3 (PMC6515611; doi:10.1186/s12889-019-6962-3)
Supplement: Supplementary file 1 — Basic information of reform county (Yong’an) and control counties (Sha and Youxi) (2013). (DOC 44 kb) [file 12889_2019_6962_MOESM1_ESM.doc]

**Additional file 1**. Basic information of reform county (Yong’an) and control counties (Sha and Youxi) (2013).

| Items | Reform county | Control counties | |
| --- | --- | --- | --- |
|  | Yong’an | Sha | Youxi |
| Population (thousands) | 348 | 228 | 353 |
| Net annual income of rural resident per capita, ¥ | 11245 | 11562 | 10691 |
| Number of village | 228 | 171 | 263 |
| % population enrolled in NCMS^a^ | 99.97 | 99.98 | 99.99 |
| Number of hospital beds (per thousand population) | 7.03 | 5.61 | 3.88 |
| Number of health care professionals (per thousand population) | 7.23 | 5.25 | 3.96 |

^a^NCMS: Rural New Cooperative Medical Scheme.
