# Supplementary material for: Cesarean delivery rates, costs and readmission of childbirth in the new cooperative medical scheme after implementation of an episode-based bundled payment (EBP) policy
Source: BMC Public Health. 2019 May 14;19:557. doi: 10.1186/s12889-019-6962-3 (PMC6515611; doi:10.1186/s12889-019-6962-3)
Supplement: Supplementary file 2 — The estimated results of difference in differences with propensity score matching. (DOC 47 kb) [file 12889_2019_6962_MOESM2_ESM.doc]

**Additional file 2**. The estimated results of difference in differences with propensity score matching

|  | Difference-in-Differences | PSMDID^a^ |
| --- | --- | --- |
| Probability of cesarean delivery | -0.4150^**^ | -0.3954^**^ |
| Ln(Total spending per admission) | -0.3146^**^ | -0.3047^**^ |
| Ln(Government expense per admission) | -0.6030^**^ | -0.6048^**^ |
| Ln(OOP^b^ payments per admission) | -0.1424^**^ | -0.1378^**^ |
| OOP^b^ payment as a share of total spending | 8.7236^**^ | 8.679^**^ |
| Ln(Drug cost per admission) | -0.8643^**^ | -0.8438^**^ |
| Ln(Diagnostic testing cost per admission) | -0.2070^**^ | -0.1909^**^ |
| Ln(Physician services and therapeutic services cost per admission) | -0.2582^**^ | -0.2486^**^ |
| Ln(Length of stay) | -0.1729^**^ | -0.1644^**^ |
| Probability of 30-day readmission | 0.4008 | 0.2976 |
| Probability of 60-day readmission | 0.2772 | 0.3706 |

^*^p＜0.05; ^**^p＜0.01. PSMDID^a^: difference in differences with propensity score matching; OOP^b^: Out-of-pocket.
